# Iatrogenic Intussusception Due to Gossypiboma Presenting as Acute Intestinal Obstruction to a Tertiary Care Emergency Department

**DOI:** 10.7759/cureus.87411

**Published:** 2025-07-07

**Authors:** Pranjul Sharma, Himanshi Baid, Harindra Sandhu, Mukta Singh

**Affiliations:** 1 Emergency Medicine, Himalayan Institute of Medical Sciences, Swami Rama Himalayan University, Dehradun, IND; 2 General Surgery, Himalayan Institute of Medical Sciences, Swami Rama Himalayan University, Dehradun, IND

**Keywords:** acute abdomen, emergency department, gossypiboma, intestinal obstruction, intussusception

## Abstract

Intussusception, typically a pediatric condition, is rare in adults and poses a diagnostic challenge in emergency settings due to its varied presentations. We present a case of a 48-year-old female with a history of recurrent abdominal pain after cholecystectomy (done two years back), who presented with an acute onset of colicky abdominal pain, vomiting, and obstipation. Initial evaluation revealed signs of intestinal obstruction and metabolic acidosis. Imaging confirmed sigmoid colo-colic intussusception with a suspected mass lesion as the lead point, later identified intraoperatively as a retained surgical mop. Despite emergent surgical intervention with bowel resection and stoma formation, the patient’s clinical course was complicated by septic shock, acute kidney injury, and refractory metabolic acidosis, leading to a fatal outcome. Adult intussusception remains a diagnostic and therapeutic challenge, often necessitating prompt surgical intervention, yet the outcome can be poor, especially in cases with significant intra-abdominal pathology and delayed presentation.

## Introduction

Acute abdomen represents about 5-10% of all adult patients coming to the emergency department [1,2]. This presentation includes a broad spectrum of diseases, including life-threatening ones, and hence, it becomes a diagnostic challenge for emergency physicians. Intussusception is a rare cause of acute abdomen in the adult age group, accounting for only about 5% of all intussusceptions, as this is commonly a pediatric age group emergency. Out of these, only about 1% account for acute bowel obstruction. Adult intussusception is seen in only 0.003-0.02% of hospital admissions [3]. The cause of intussusception in adults is usually secondary to gastrointestinal tumors (both benign and malignant) acting as a lead point [3]. Only a few cases where iatrogenic mechanical factors caused intussusception-after removal of a long intestinal tube [4] or a jejunal feeding tube acting as the lead point [5]-have been reported. However, to the best of our knowledge, only one case of gossypiboma acting as a lead point for iatrogenic intussusception has been reported in the literature so far [6].

## Case presentation

An Asian female in her 40s presented to our ED, an urban tertiary care hospital, with complaints of colicky abdominal pain for 15 days associated with vomiting (non-bilious, non-projectile, non-blood-tinged). She subsequently developed obstipation two days before ED presentation, with further episodes of vomiting, and began to feel unwell. She was unable to tolerate even oral fluids, which prompted her presentation to the ED. She had a history of multiple hospital admissions for similar complaints in the past one and a half years, where she was managed conservatively for subacute intestinal obstruction. She had a previous history of open cholecystectomy two years ago.

**Figure 1:**
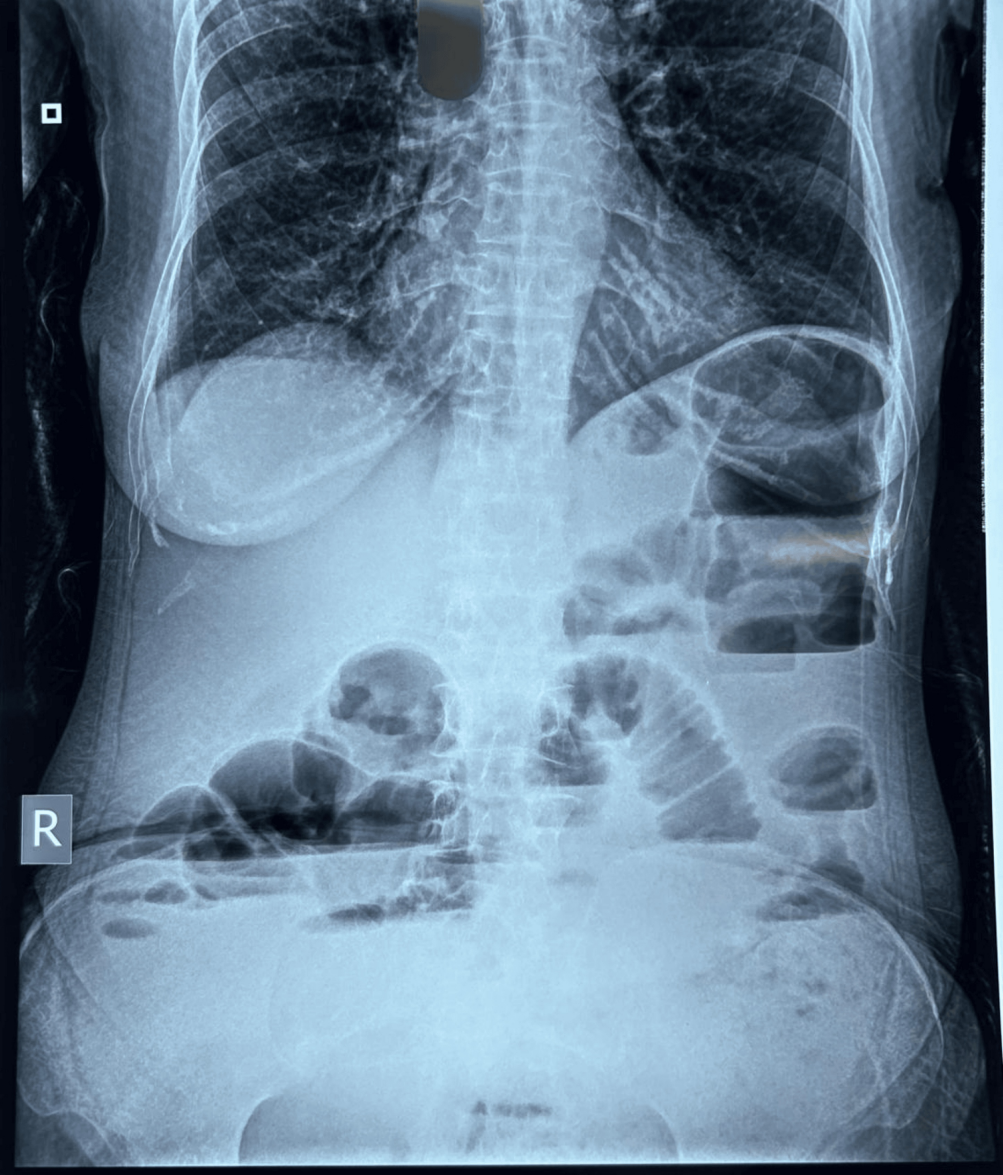
Abdominal X-ray of the patient showing multiple air-fluid levels suggestive of acute intestinal obstruction

The primary assessment of the patient was unremarkable except for tachypnea and tachycardia (respiratory rate of 22/min, heart rate of 110/min) on arrival. The patient was shifted to a monitored bed. Abdominal examination revealed a tense and distended abdomen with generalized tenderness. There was no palpable mass, and the rectal examination did not demonstrate any blood. Bowel sounds were sluggish, with no signs of peritonitis. Intravenous access was taken, and blood investigations were carried out. The patient was given pain relief (with IV paracetamol 1 g), a proton pump inhibitor, antiemetics, and maintenance fluid management with Ringer's lactate (at the rate of 100 ml/hour). Venous blood gas revealed a metabolic acidosis (pH 7.42, pCO₂ 26.7 mmHg, pO₂ 54.5 mmHg, HCO₃ 17.1 mmol/L, base excess -6.1 mmol/L, lactate 0.8 mmol/L). Other blood tests illustrated low hemoglobin (7.40 g/dl), raised white cell counts (16.20 thou/cu mm), a neutrophil count of 52.40%, and an initial creatinine of 2.01 mg/dl. The abdominal X-ray of the patient revealed multiple air-fluid levels suggestive of acute intestinal obstruction, as shown in Figure 1.

**Figure 2:**
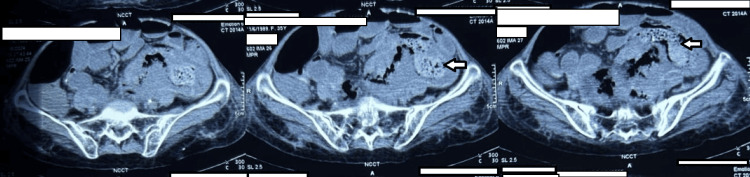
A non-contrast CT of the abdomen showing sigmoid colo-colic intussusception with an ill-defined lesion noted in the intussuscipiens. Panel 1: Cross-section showing sigmoid colo-colic intussusception. Panel 2: The arrow points at the ill-defined lesion seen in the intussuscipiens. Panel 3: The arrow points at the ill-defined lesion seen in the intussuscipiens.

A non-contrast CT of the abdomen was then performed, which revealed sigmoid colo-colic intussusception with an ill-defined mass lesion noted in the intussuscipiens, with resultant acute intestinal obstruction, as depicted in Figure 2.

**Figure 3:**
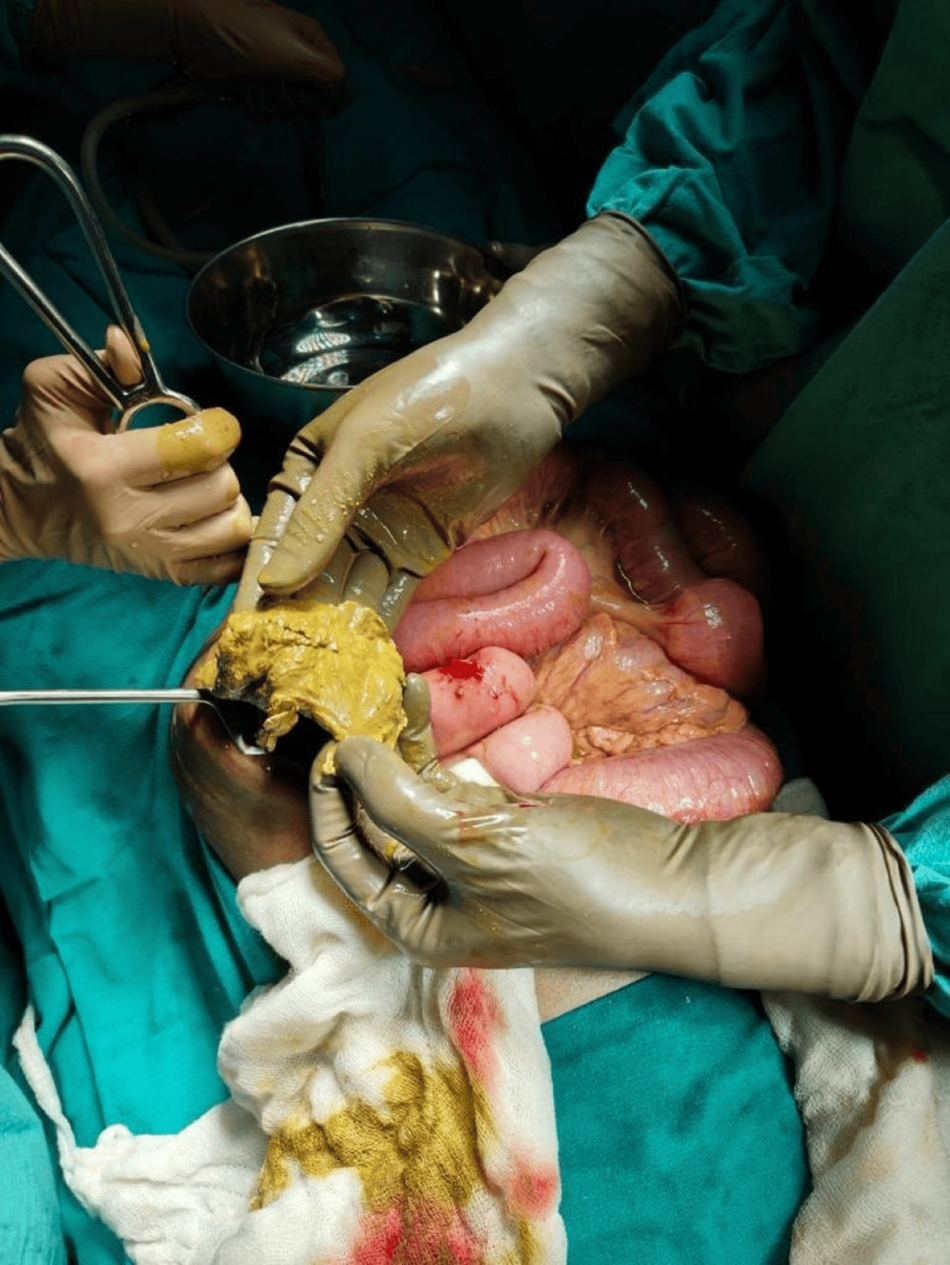
Intraoperative image showing the retained gauze retrieved during surgery

A surgical consult was taken for the patient, and she was taken for emergency explorative laparotomy. The patient was transfused with two units of PRBC and given a loading dose of the antibiotic piperacillin-tazobactam (4.5 g IV) preoperatively. Preoperatively, after the resuscitative measures, the patient's heart rate was still 110/min, RR was 20/min, and blood pressure was 112/70 mmHg; however, the patient had no urine output. She underwent exploratory laparotomy with double-barrel ileostomy and loop colostomy. Intraoperatively, approximately 50-100 ml of feculent material was present in the peritoneal cavity, and multiple intra-bowel adhesions were present within the bowel loops. A gauze piece (mop), probably from past surgery, was recovered from inside the peritoneal cavity that was adhered to the descending colon, through which the proximal ileum (approximately 100 cm from the distal jejunum) was adhered to the descending colon, as depicted in Figure 3.

There was a large, perforated segment at the descending colon for which a segment was taken out as a loop colostomy, and a pelvic drain was placed. She was transferred to the intensive care unit postoperatively for optimization. However, on post-operative day (POD)-2, the patient worsened clinically, developing shock for which she was taken on ventilatory and vasopressor support. The patient's general condition further deteriorated with worsening refractory metabolic acidosis due to the development of acute kidney injury. Two cycles of sustained low-efficiency dialysis were also done. But the patient deteriorated further and finally collapsed on POD-4, and despite best efforts, the patient succumbed.

## Discussion

Intussusception is defined as the invagination of one segment of the bowel into an immediately adjacent segment of the bowel. It is infrequent, found in less than 1 in 1300 abdominal operations. The pediatric population is afflicted more than adults, with an approximate ratio of 20:1 [7]. In contrast to intussusception in children, which is typically primary or idiopathic, most adult intussusception is caused by abnormal growths in the intestines, such as benign and cancerous tumors and polyps, or abdominal adhesions that are formed after surgery or diseases like cystic fibrosis, celiac disease, and Crohn's disease [3].

Abdominal pain, nausea, and vomiting are the predominant symptoms in the majority of patients diagnosed with intussusception. Symptoms typically endure for an extended period, though there are instances where the patient may suddenly experience severe abdominal pain [8]. In our case, the patient presented with abdominal pain for over 15 days, which further escalated to obstipation two days before presentation. It is commonly understood that when there are growths or obstructions in the bowel, they can irritate the lining and trigger abnormal contractions, possibly causing intussusception. The same effect of mechanical irritation can be extended to the few cases noted of iatrogenic intussusception, although the exact mechanism is still not precise. Adult intussusception occurs mainly in the small bowel and is classified on the basis of location. It can be categorized as entero-enteric (small bowel only), colo-colic (large bowel only), ileocolic (terminal ileum prolapses within the ascending colon), or ileocecal (ileocecal valve is the lead point) [9]. Ultrasonography is frequently employed in suspected cases of intussusception due to its clarity, accessibility, and non-invasiveness. Ultrasound demonstrates a preoperative diagnostic accuracy of 78.5%. In circumstances where there is a palpable abdominal mass, ultrasonography diagnostic accuracy improves to 86.6% [10]. In this scenario, the patient underwent multiple ultrasound examinations, all indicating the presence of subacute intestinal obstruction, until the patient attended our institution for acute intestinal obstruction. Repeated diagnostic processes due to the low sensitivity of ultrasound and the rarity of adult intussusception resulted in a delay in correct and prompt diagnosis, and the patient sought clinical help from various primary and secondary level facilities before attending our emergency department at tertiary level care. Currently, abdominal CT scans are the most effective imaging tool for detecting intussusception, with diagnostic accuracy ranging from 58-100% [11]. The typical image shows a thickened intestinal wall and mesentery within the lumen, resembling the "target sign" or "sausage-shaped" appearance in axial projection [3]. A CT scan pinpoints the precise location of intussusception and detects signs of potential intestinal nonviability. However, it may not always distinguish between a pathological lesion and a thickened edema of the intestinal wall, except in cases of lipoma [7]. As discussed above, the CT abdomen confirmed the diagnosis of colo-colic intussusception with an ill-defined mass lesion noted in the intussuscipiens with resultant acute intestinal obstruction. However, it was only during the exploratory laparotomy that this mass was revealed as a gossypiboma.

A retained foreign body should be considered in the differential diagnosis of any postoperative patient presenting with pain, infection, or a palpable mass. The low index of suspicion is usually due to the rarity of the condition and the latency in the manifestation. The history of previous surgery is mandatory for the diagnosis of gossypiboma [12]. The legal implications of gossypiboma are high, as the condition is associated with morbidity and mortality. Again, gossypiboma may be misdiagnosed as a malignant tumor, leading to unnecessary invasive investigations and extirpative surgery, which may be disabling [13]. In this instance, the patient underwent cholecystectomy approximately two years ago, which could be the likely reason for the presence of a gauze piece (mop) inside the peritoneal cavity. The CT scan also only identified it as an ill-defined mass, as a definitive diagnosis of gossypiboma can be made only in the presence of a radio-opaque marker, which is usually not present on surgical gauze [6].

Sponge migration typically happens in the small intestine and can result in partial obstruction of the small bowel, particularly when it migrates to the ileocecal valve. The lack of severe bowel blockage prior to the intussusception in this instance may be explained by straight migration to the colon. The same was also observed in the case reported earlier [6]. In adults with intussusception and retained foreign bodies, treatment typically involves surgery and usually involves removing the affected part of the bowel, followed by primary anastomosis. Prognosis is generally poor in adult intussusception due to delayed presentation, identification, and involvement of malignancy [6]. Our patient underwent exploratory laparotomy with double-barrel ileostomy loop colostomy within hours after her presentation to the ED; however, she succumbed due to the delayed diagnosis from the onset of her symptoms.

Although we could not save this patient, we would like to bring forth this rare case so that a high index of suspicion is maintained, especially by the emergency physicians (the usual first contacts with such patients), in patients who have undergone past surgeries for the rare complications due to retained sponges and also of intussusception in adults so that they can be diagnosed at the very beginning of the symptoms and not after they have developed complications like acute intestinal obstruction, as was seen in our case.

## Conclusions

Intussusception, typically a pediatric condition, is rare in adults and poses a diagnostic challenge in emergency settings due to its varied presentations. Through our case, we presented one such rare clinical scenario where adult intussusception was repeatedly misdiagnosed for nearly two years. This was detected only when the patient presented with the complication of acute intestinal obstruction. Although this patient could not be saved despite best efforts, we would urge the frontline physicians and surgeons to maintain a high index of suspicion in patients who have undergone past surgeries for the rare complications due to retained sponges and also of intussusception in adults so that they can be diagnosed at the very beginning of the symptoms and not after they have developed complications like acute intestinal obstruction, as was seen in our case.
